# Effects of Microenvironmental Changes on the Fluorescence Signal of Alternariol: Magnesium Induces Strong Enhancement in the Fluorescence of the Mycotoxin

**DOI:** 10.3390/ijms22168692

**Published:** 2021-08-13

**Authors:** Eszter Fliszár-Nyúl, Beáta Lemli, Sándor Kunsági-Máté, Miklós Poór

**Affiliations:** 1 Department of Pharmacology, Faculty of Pharmacy, University of Pécs, Rókus u. 2, H-7624 Pécs, Hungary; eszter.nyul@aok.pte.hu; 2 Food Biotechnology Research Group, János Szentágothai Research Centre, University of Pécs, Ifjúság útja 20, H-7624 Pécs, Hungary; 3 Department of Organic and Pharmacological Chemistry, Faculty of Pharmacy, University of Pécs, Szigeti út 12, H-7624 Pécs, Hungary; beata.lemli@aok.pte.hu (B.L.); sandor.kunsagi-mate@aok.pte.hu (S.K.-M.); 4 Green Chemistry Research Group, János Szentágothai Research Centre, University of Pécs, Ifjúság útja 20, H-7624 Pécs, Hungary

**Keywords:** alternariol, mycotoxin, fluorescence, microenvironment, magnesium, fluorescence enhancers, high-performance liquid chromatography

## Abstract

Alternariol (AOH) is an emerging mycotoxin produced by *Alternaria* molds. It occurs as a contaminant e.g., in oilseeds, cereals, grapes, and tomatoes. Chronic exposure to AOH may cause genotoxic and endocrine disruptor effects. Our recent studies demonstrated that the fluorescence signal of AOH can be strongly affected by the environmental pH as well as by the presence of serum albumin or cyclodextrins. In the current study, we aimed to characterize the most optimal circumstances regarding the highly sensitive fluorescent detection of AOH. Therefore, the further detailed investigation of the microenvironment on the fluorescence signal of the mycotoxin has been performed, including the effects of different buffers, organic solvents, detergents, and cations. Organic solvents (acetonitrile and methanol) caused only slight increase in the emission signal of AOH, while detergents (sodium dodecyl sulfate and Triton-X100) and Ca^2+^ induced considerably higher enhancement in the fluorescence of the mycotoxin. In addition, Mg^2+^ proved to be a superior fluorescence enhancer of the AOH. Spectroscopic and modeling studies suggest the formation of low-affinity AOH-Mg^2+^ complexes. The effect of Mg^2+^ was also tested in two HPLC assays: Our results show that Mg^2+^ can considerably increase the fluorescence signal of AOH even in a chromatographic system.

## 1. Introduction

**Figure 1:**
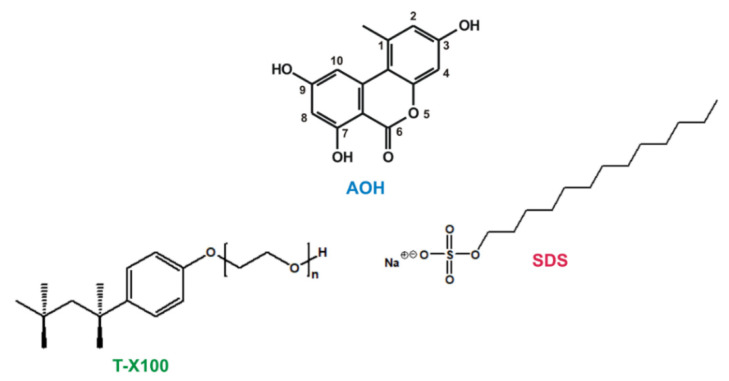
Chemical structures of alternariol (AOH), Triton-X100 (T-X100), and sodium dodecyl sulfate (SDS).

*Alternaria* molds commonly appear in cereals, oilseeds, tomatoes, citrus fruits, and grapes; they can produce several toxic secondary metabolites (including alternariol, alternariol monomethyl ether, tentoxin, tenuazonic acid, altenuene, and altertoxins) which can be classified as phyto- and mycotoxins [1]. Alternariol (AOH, Figure 1) is a dibenzo-α-pyrone *Alternaria* mycotoxin. Its acute toxicity is considered to be low compared to e.g. aflatoxin B1 or ochratoxin A [2,3,4]. However, the chronic exposure to AOH can cause mutagenic, genotoxic, immunotoxic, and/or endocrine disruptor effects [2,5]. Despite the fact that AOH is an emerging mycotoxin which raises increasing concerns regarding its health effects, no regulatory limits have been established on its maximal threshold concentrations in food, animal feed, and beverages [6,7]. The relative level of concern of AOH for human health was expressed by the European Food Safety Authority (EFSA) as threshold of toxicological concern, and its value for AOH is 2.5 ng/kg bodyweight [7]. Nevertheless, the study of EFSA also indicated that the dietary exposure to this mycotoxin frequently exceeds the threshold suggested. Furthermore, there is still an urgent demand for the further data collection regarding the occurrence and toxicity of AOH as well as its metabolites for the proper risk assessment [8]. Since AOH exerts fluorescence, its molecular interactions can be successfully investigated and characterized employing fluorescence spectroscopy [9,10], and it can be quantified by HPLC-FLD (high-performance liquid chromatography coupled to fluorescence detector) technique [6,11]. Our recent studies demonstrated that the environmental pH can strongly affect the fluorescence spectra of AOH; in addition, serum albumin and cyclodextrins can considerably increase the fluorescence signal of the mycotoxin [9,10]. These observations highlight that microenvironmental changes have high importance regarding the fluorescence detection of AOH.

The microenvironment (e.g., the pH, ionic strength, polarity and viscosity of the solvent, detergents, or metal ions) can commonly influence the absorption and fluorescence spectra of optically active compounds, including certain mycotoxins [12]. Zearalenone and zearalenol can be detected with higher sensitivity with HPLC-FLD as a result of the post-column derivatization using aluminum chloride [13]. Furthermore, surfactants such as sodium dodecyl sulfate (SDS, Figure 1) and Triton-X100 (T-X100, Figure 1) effectively increased the emission signal of zearalenone [14]. In another study, T-X100 also strongly enhanced the fluorescence of aflatoxins (B1, B2, G1, and G2), allowing the development of a highly sensitive spectrofluorimetric method for their quantification from wheat samples (after magnetic solid phase extraction) [15]. Complex formation with aluminum ion markedly increased the fluorescence of citrinin, while the emission intensity of another mycotoxin, ochratoxin A was only slightly affected under the same conditions [12]. Nevertheless, ochratoxin A forms complexes with other metal ions, including lithium, calcium, magnesium, and zinc [16,17]. Moreover, the addition of Mg^2+^ to the eluent caused a two-fold increase in the sensitivity of the HPLC-FLD method used, due to the magnesium-induced elevation in the emission signal of ochratoxin A [17].

In this study, we aimed to investigate the effects of microenvironmental changes on the fluorescence emission signal of AOH, which helps to understand which conditions favor the fluorescence detection of the mycotoxin. Therefore, the influence of environmental pH, organic solvents, cations, and detergents has been examined. Our results demonstrate that magnesium ions can cause the highest increase in the emission signal of AOH, which is superior even vs. solvents and detergents tested. Modeling studies were also performed to characterize AOH-Mg^2+^ interaction. In addition, based on the strong fluorescence signal of the AOH-Mg^2+^ complex, two HPLC-FLD methods have been optimized and demonstrated, which highlights that magnesium can strongly increase the sensitivity of fluorescence based analytical detection of AOH.

## 2. Results and Discussion

### 2.1. Effect of the Environmental pH on the Fluorescence Spectra of AOH

In our previous study, the excitation and emission spectra of AOH were recorded at pH 5.0, 7.4, and 10.0, showing that the ionization of the molecule strongly modifies its fluorescence [10]. In the current study, for the more detailed characterization regarding the influence of the pH, fluorescence spectra of AOH were tested at pH 2.0, 4.0, 6.0, 8.0, and 10.0.

**Figure 2:**
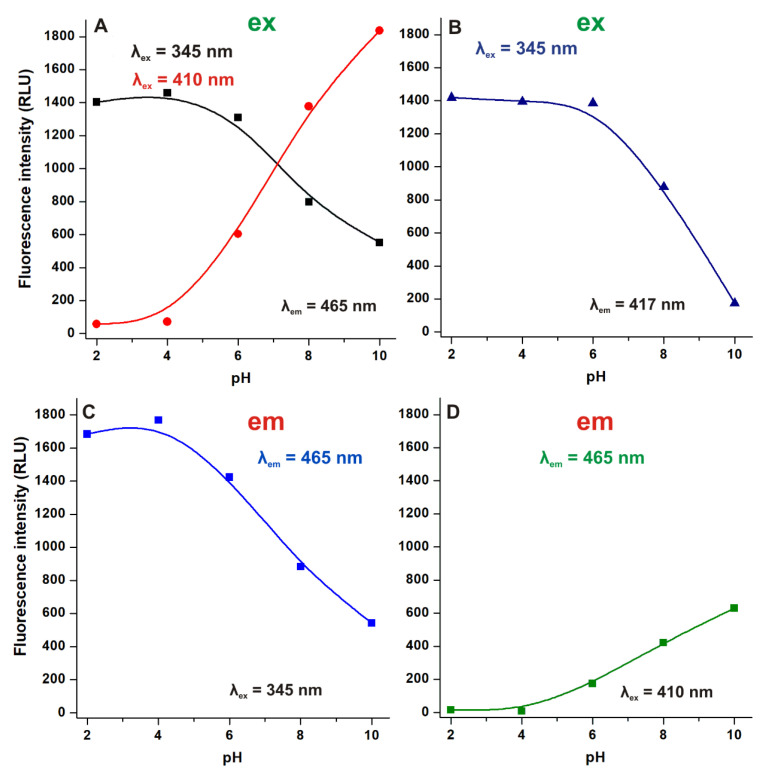
Excitation ((**A**,**B**): ex and em slits were 10 nm) and emission (**C**): ex and em slits were 10 nm; (**D**): ex and em slits were 5 nm and 10 nm, respectively signals of AOH (25 μM) in sodium phosphate buffers (pH 2.0, 4.0, 6.0, 8.0, and 10.0; RLU: relative light unit).

Using 465 nm emission wavelength: (1) an excitation peak can be noticed at 345 nm, its intensity is gradually decreasing with the elevation of the pH (above pH 4.0); (2) while at pH 6.0 a second excitation peak appears at 410 nm, which is increasing with the further elevation of the pH (Figure 2A). However, applying 417 nm emission wavelength, we can notice a gradual decrease in the excitation signal at 345 nm above pH 6.0 (Figure 2B). Based on our previous study [10], the excitation peak at 345 nm is presented by both non-ionized and ionized mycotoxin molecules, while the excitation peak at 410 nm belongs to the ionized form of AOH.

When 345 nm excitation wavelength was applied, one dominant emission peak was observed at 465 nm, which gradually decreased above pH 4.0 (Figure 2C). However, employing 410 nm excitation wavelength, an emission peak at 465 nm appeared only at pH 6.0 and increased with the further elevation of the pH (Figure 2D). These observations are also in agreement with our previous study [10]: (1) the 345 nm excitation wavelength is suitable to excite both non-ionized and ionized AOH, nevertheless, the ionized form exerts lower fluorescence under these conditions; (2) the 410 nm excitation wavelength belongs to the ionic form(s) of AOH.

Since AOH shows emission signal in a wide pH range using 345 nm excitation, in the following experiments, we recorded the emission spectra of the mycotoxin using this wavelength. Furthermore, we applied acidic or neutral conditions because at higher pH, the deprotonation of the mycotoxin results in the appearance of several ionization forms and tautomers [10] which makes the interpretation of data extremely difficult.

### 2.2. Effects of Acetonitrile and Methanol on the Emission Signal of AOH

**Figure 3:**
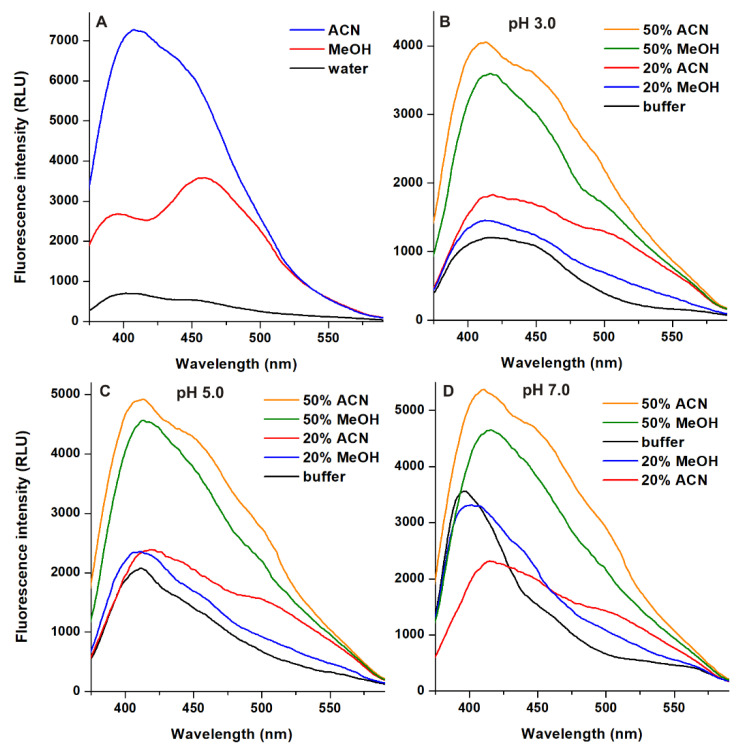
Emission spectra of AOH (25 μM) in pure solvents ((**A**); ex slit: 5 nm, em slit: 10 nm) and in the presence of 20 or 50 *v/v*% organic solvents in sodium phosphate ((**B**); 50 mM, pH 3.0; ex and em slits: 10 nm), sodium acetate ((**C**); 50 mM, pH 5.0; ex and em slits: 10 nm), and Tris-HCl ((**D**); 50 mM, pH 7.0; ex and em slits: 10 nm) buffers (λ_ex_ = 345 nm; ACN, acetonitrile; MeOH, methanol; RLU, relative light unit).

In reversed phase HPLC systems, typically acetonitrile and methanol are applied as organic eluent components, therefore, fluorescence emission spectra of AOH (λ_ex_ = 345 nm) were recorded in water, methanol, and acetonitrile as well as in the binary mixtures of these organic solvents with aqueous buffers. AOH showed a wide emission peak in water (which can be composed of two peaks, likely belonging to the two tautomeric forms of nonionic AOH), as it has also been reported [10]. However, much higher emission signals of the mycotoxin were noticed in acetonitrile and methanol, with the emission wavelength maxima at 405 and 455 nm, respectively (Figure 3A).

The effects of acetonitrile and methanol on the emission signal of AOH (λ_ex_ = 345 nm) were also examined at pH 3.0, 5.0, and 7.0 (Figure 3). Only a slight increase in the fluorescence of the mycotoxin was caused by 20 *v*/*v*% acetonitrile and methanol at pH 3.0 and 5.0, while these solvent concentrations decreased the emission signal at pH 7.0. However, the presence of 50 *v*/*v*% acetonitrile and methanol caused approximately 1.5- to 3-fold increase in the emission signal of AOH (Figure 3).

These data suggest that higher acetonitrile and methanol concentrations lead to the stronger fluorescence enhancement of AOH, and the aprotic solvent acetonitrile enhances the emission signal better compared to methanol. The stronger fluorescence of AOH in acetonitrile and methanol (vs. water) can be explained by the lower quenching effects of water molecules in the solvation shell [18,19], as it has also been demonstrated in the presence of cyclodextrins [10].

### 2.3. Effects of Detergents on the Emission Signal of AOH

**Figure 4:**
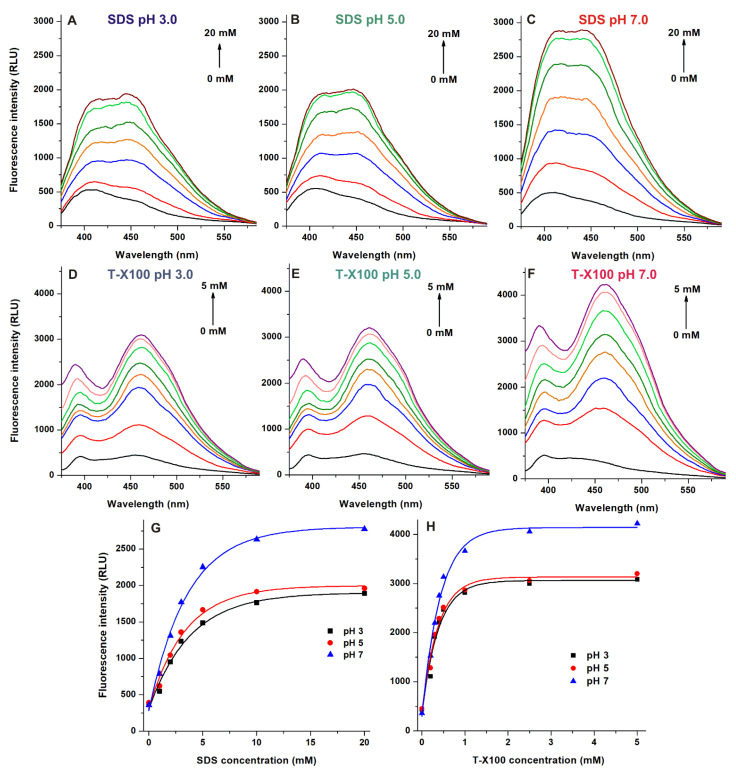
Fluorescence emission spectra of AOH (5 μM) in the presence of increasing concentrations of sodium dodecyl-sulfate (SDS; pH 3.0 (**A**), pH 5.0 (**B**), and pH 7.0 (**C**) and Triton-X100 (T-X100); pH 3.0 (**D**), pH 5.0 (**E**), and pH 7.0 (**F**). SDS- (**G**) and T-X100-induced (**H**) increase in the emission signal of AOH (λ_ex_ = 345 nm, λ_em_ = 460 nm; ex and em slits: 10 nm; buffers used: 50 mM pH 3.0 sodium phosphate, 50 mM pH 5.0 sodium acetate, and 50 mM pH 7.0 Tris-HCl; RLU: relative light unit).

Micellar encapsulation of fluorophores can result in fluorescence enhancement or quenching due to the formation of an altered microenvironment inside the micelles [14,20]. Therefore, in our following experiment, we investigated the interaction of AOH with surfactants at pH 3.0, 5.0, and 7.0. Emission spectra of the mycotoxin (5.0 μM; λ_ex_ = 345 nm) were recorded in the presence of sodium dodecyl sulfate (SDS; anionic detergent) and Triton-X100 (T-X100; nonionic detergent). Both SDS and T-X100 induced concentration dependent increase in the fluorescence of AOH, with two visible components of the emission spectra (Figure 4). Same phenomenon was noticed previously in the presence of cyclodextrins [10]. Detergent-induced changes in the fluorescence intensity were similar at pH 3.0 and pH 5.0, while both SDS and T-X100 induced higher increase in the emission signal of AOH at pH 7.0 (Figure 4). Regardless of the environmental pH, T-X100 proved to be a better fluorescence enhancer vs. SDS (T-X100: 7.7-fold at pH 3.0, 7.0-fold at pH 5.0, and 9.9-fold at pH 7.0; SDS: 4.9-fold at pH 3.0, 5.0-fold at pH 5.0, and 8.4-fold at pH 7.0). In accordance with the results regarding zearalenone-detergent interactions [14], the greater micelle size and the lower CMC (critical micelle concentration) value of T-X100 in aqueous solution can explain our observations. Furthermore, T-X100 also slightly increased the fluorescence of aflatoxins after their extraction from wheat samples [15].

### 2.4. Effects of Cations on the Emission Signal of AOH

**Figure 5:**
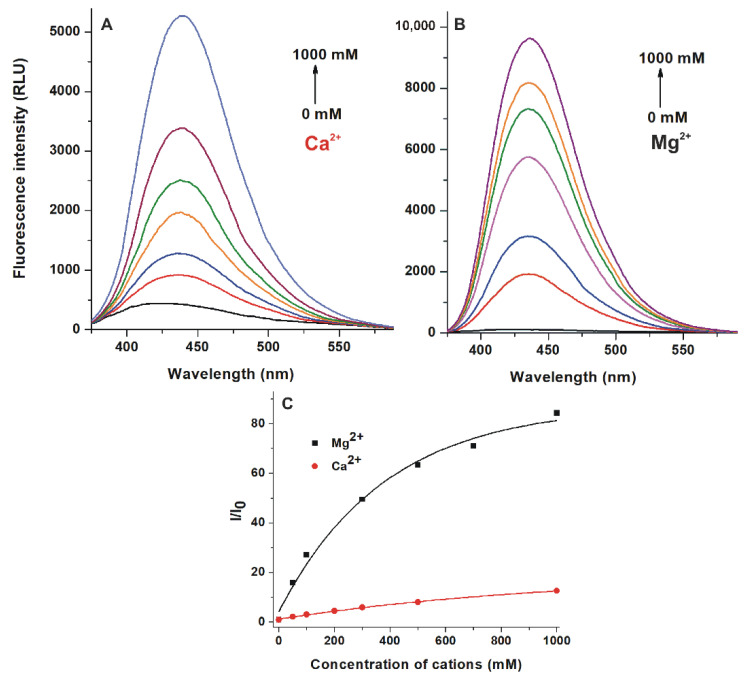
Fluorescence emission spectra of AOH (5 μM) in the presence of increasing Ca^2+^ ((**A**); ex and em slits: 10 nm) and Mg^2+^ ((**B**); ex slit: 5 nm, em slit: 10 nm) concentrations in Tris-HCl buffer (50 mM, pH 7.0). Cation-induced relative increase in the emission signal of AOH ((**C**); λ_ex_ = 345 nm, λ_em_ = 440 nm; RLU: relative light unit).

Emission spectra of AOH were recorded (λ_ex_ = 345 nm) in the presence of different cations, including alkali metal ions (Na^+^ and K^+^), alkaline earth metal ions (Mg^2+^, Ca^2+^, and Ba^2+^), and transitional metal ions (Zn^2+^, Cu^2+^, and Fe^2+^) in Tris-HCl buffer (50 mM, pH 7.0). Na^+^, K^+^, Ba^2+^, Zn^2+^, Cu^2+^, and Fe^2+^ did not affect or barely modified the emission spectra of the mycotoxin (data not shown). However, in a concentration-dependent fashion, Ca^2+^ and Mg^2+^ markedly increased the fluorescence signal of AOH (Figure 5), suggesting the interaction of these cations with the mycotoxin. Ca^2+^ (1000 mM) produced 12-fold while Mg^2+^ (1000 mM) caused approximately 80-fold elevation in the emission intensity of the mycotoxin at 440 nm (emission wavelength maxima of the mycotoxin-cation complexes formed) (Figure 5C).

Based on the fluorescence emission data (Figure 5C), binding constants (*K*) of AOH-Ca^2+^ and AOH-Mg^2+^ complexes were determined employing the Hyperquad2006 software and the Benesi-Hildebrand equation. Table 1 demonstrates *K* (L/mol) values calculated using the two evaluations, showing that both complexes have low stability. Furthermore, Mg^2+^ is not only a better fluorescence enhancer but it forms more (approximately four-fold) stable complex with AOH compared to Ca^2+^. Similar observations were published in an earlier study regarding the interactions of ochratoxin A with alkali and alkaline earth metal ions; however, ochratoxin A binds to these cations with much higher affinity [16].

**Figure 6:**
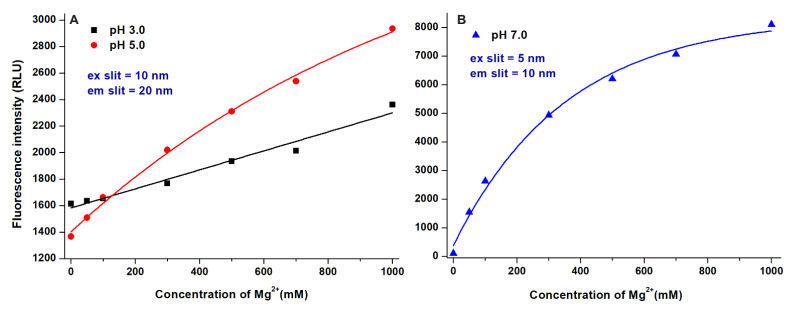
Effects of increasing Mg^2+^ concentrations on the fluorescence emission signal of AOH (5 μM) in sodium phosphate ((**A**); 50 mM, pH 3.0; ex slit: 10 nm, em slit: 20 nm), sodium acetate ((**A**); 50 mM, pH 5.0; ex slit: 10 nm, em slit: 20 nm), and Tris-HCl ((**B**); 50 mM, pH 7.0; ex slit: 5 nm, em slit: 10 nm) buffers (λ_ex_ = 345 nm, λ_em_ = 440 nm; RLU: relative light unit).

AOH-Mg^2+^ interaction was further examined under acidic conditions (pH 3.0 and 5.0). Mg^2+^ caused concentration-dependent but moderate increase in the emission signal of AOH at pH 3.0 and pH 5.0 compared to the data observed at pH 7.0 (Figure 6). Interestingly, the further increase in the pH (pH 10.0 was tested; data not shown) caused lower fluorescence enhancement and binding constant than at pH 7.0, suggesting that Mg^2+^ may prefer the monoanionic form of AOH. This hypothesis is also supported by modeling studies (see in Section 2.5).

The increase in the fluorescence signal of AOH in the presence of Ca^2+^ and Mg^2+^ can be explained by two mechanisms: (1) the nonradiative dissipation of the energy from the excited state is inhibited by the slower molecular motion of the more rigid structure (stabilized by the cation-molecule bonds); (2) the disruption of the hydration shell around the mycotoxin decreases the partial quenching effects of water molecules, resulting in the higher fluorescence of AOH-cation complexes. The former effect enhances the fluorescence quantum yield [21] while the latter impact reduces the quenching effects of water molecules on the aromatic fluorophore [22]. The latter phenomenon is supported by modeling studies: Mg^2+^ ions show higher binding constants than Ca^2+^ toward both the nonionic and monoanionic forms of AOH (Table 2), which is associated with the extensive removal of water molecules from the hydration shell, and causing a larger enhancement in the emission intensity.

### 2.5. Investigation of AOH-Mg^2+^ and AOH-Ca^2+^ Interactions Employing Molecular Modeling Studies

**Figure 7:**
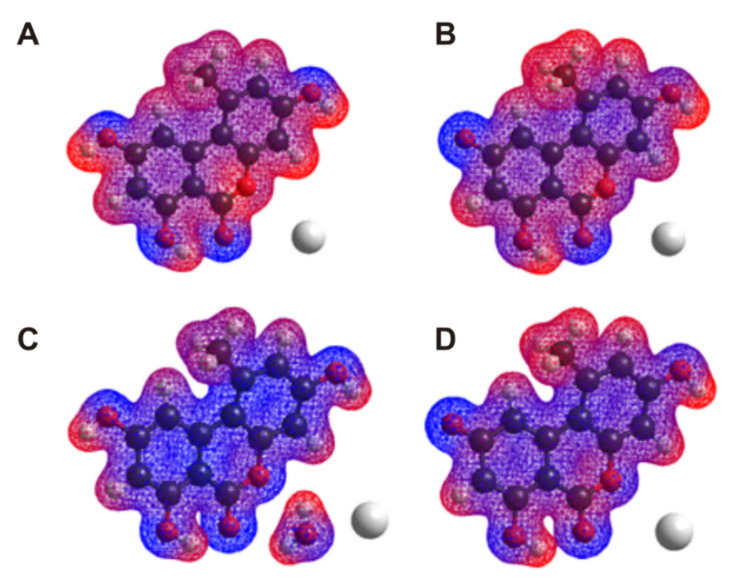
Electrostatic potential map associated to the most stable equilibrium conformations of AOH-cation complexes: nonionic AOH-Mg^2+^ (**A**), monoanionic AOH-Mg^2+^ (**B**), nonionic AOH-Ca^2+^ (**C**), and monoanionic AOH-Ca^2+^ (**D**).

Interaction of the nonionic and monoanionic forms of AOH with Mg^2+^ and Ca^2+^ were examined in aqueous environment. Calculated binding constants of these complexes are demonstrated in Table 2. Our results suggest that AOH forms complexes with the selected cations (Figure 7). Higher *K* values were obtained for the AOH-Mg^2+^ compared to the AOH-Ca^2+^ complexes. Monoanionic AOH forms more stable complexes with the cations than the nonionic AOH, reflecting the involvement of Coulomb forces in the molecular interactions. Considering the aqueous environment and the weak interactions, competitive processes can occur between the hydration and the AOH-cation interactions. Typically, the interacting molecules lose their hydration shell prior their primary interactions. However, during the interaction of the nonionic form of AOH with Ca^2+^, the removal of the last water molecule from the hydration shell of Ca^2+^ requires higher energy than the energy gain of the complex formation. Therefore, AOH-Ca^2+^ complex will be formed through the microsolvation of Ca^2+^ ion. Thus, the determinant forces of the complex formation are offered by hydrogen bonds, which are much weaker than the Coulomb forces appearing during the direct interaction of AOH molecules and the completely dehydrated Ca^2+^ ions. These observations are in agreement with a previous study which also demonstrated that higher hydration energy of Ca^2+^ is observed when the hydration shell composed less than six water molecules compared to the hydration energy of the Mg^2+^ [23].

### 2.6. Probing the Analytical Application of AOH-Mg^2+^ Interaction to Increase the Sensitivity of HPLC-FLD Methods

**Figure 8:**
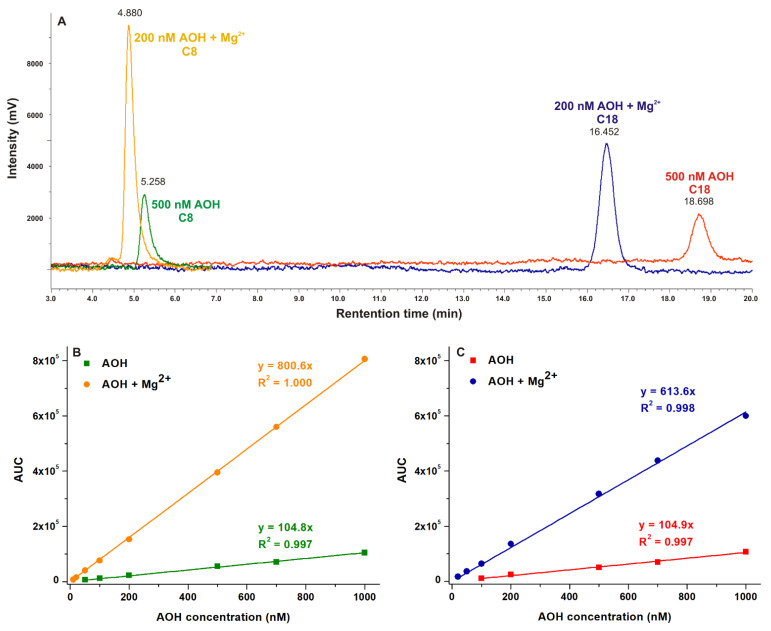
Representative chromatograms of AOH without or with MgCl_2_ (50 mM) in the eluent (**A**). Linearity of the concentration-AUC curves regarding the C8 (**B**) and C18 (**C**) methods.

Based on the results of fluorescence spectroscopic studies, Mg^2+^ markedly increased the emission signal of AOH at pH 7.0 (Figure 6). Since the fluorescence enhancer effect of Mg^2+^ on AOH did not decrease even in the presence of 30% acetonitrile (data not shown), it was reasonable to hypothesize that AOH-Mg^2+^ interaction may be suitable to increase the sensitivity of HPLC-FLD methods. We applied two different (C8 and C18 types) analytical columns for the evaluations, using the same eluent (10 mM pH 7.0 HEPES buffer and acetonitrile (70:30 *v*/*v*%), without or with 50 mM MgCl_2_) and conditions (see detailed in 3.4). In both methods tested, Mg^2+^ slightly shortened the retention time of the mycotoxin and considerably increased the height and the area of its chromatographic peaks (Figure 8). Mg^2+^ ions caused approximately six- to sevenfold signal amplification of AOH, without increasing the baseline noise. Each method (C8 and C18, with or without Mg^2+^) showed good linearity (R^2^ = 0.997 to 1.000) in the 0–1000 nM concentration range (Figure 8B,C). Limit of detection (LOD) and limit of quantification (LOQ) data determined are demonstrated in Table 3. Intra-day repeatability was also examined for the Mg^2+^-sensitized methods, it was 4.1% and 1.8% for the analyses performed using C8 and C18 columns, respectively.

The analytical quantification of *Alternaria* toxins, including AOH, can be performed employing various chromatographic techniques, such as thin layer chromatography, gas chromatography, HPLC, and liquid chromatography coupled with mass spectrometry [6]. The sensitivity of these methods highly depends on the sample matrix. The LOD and LOQ values of the recent (HPLC or LC-MS/MS) methods are typically in the range of 0.01–6.0 μg/kg and 0.03–12.0 μg/kg, respectively [6]. Our Mg^2+^-enhanced methods proved to be more sensitive than the HPLC-FLD method reported by Rico-Yuste et al. (LOD = 7.0 μg/kg, LOQ = 20.0 μg/kg) [24]. In addition, the sensitivity of the Mg^2+^-enhanced C8 method is only slightly lower compared to certain LC-MS/MS methods previously reported regarding the measurement of AOH from wheat (LOD = 0.75 μg/kg, LOQ = 2.5 μg/kg) [25] and tomato-based products (LOD = 1.4 μg/kg, LOQ = 3.5 μg/kg) [26]. Therefore, the application of Mg^2+^ seems to be a cost-effective tool to significantly increase the sensitivity regarding the fluorescence based analytical detection of AOH.

## 3. Materials and Methods

### 3.1. Reagents

Alternariol (AOH) was purchased from Cfm Oskar Tropitzsch GmbH (Marktredwitz, Germany). 4-(2-hydroxyethyl)-1-piperazineethanesulfonic acid (HEPES) and Triton-X100 (T-X100) were obtained from Merck (Darmstadt, Germany). Sodium dodecyl sulfate (SDS), and tris(hydroxymethyl)-aminomethane (Tris) were purchased from Reanal (Budapest, Hungary). HPLC grade acetonitrile was obtained from VWR (Debrecen, Hungary). Stock solution of AOH (5000 μM) was prepared in dimethyl sulfoxide (DMSO, spectroscopic grade; Fluka, NJ, US) and stored at −20 °C, protected from light.

### 3.2. Fluorescence Spectroscopic Measurements

Fluorescence spectroscopic measurements were performed employing a Hitachi F-4500 spectrofluorimeter (Tokyo, Japan) at 25 °C, in the presence of air. Background correction was carried out in each experiment.

The effect of environmental pH on the spectroscopic properties of AOH was tested in 50 mM phosphate buffers (pH 2.0, 4.0, 6.0, 8.0, and 10.0), the pH was tuned by sodium hydroxide (1 M). Excitation spectra of the mycotoxin (20 μM) were recorded employing 417 and 465 nm emission wavelengths, while emission spectra were obtained using 345 and 410 nm excitation wavelengths.

We also examined the emission spectra of AOH in water, acetonitrile, and methanol as well as in the binary mixtures of methanol or acetonitrile with aqueous buffers. Emission spectra of AOH (25 μM) were recorded in the presence of 0, 20, and 50 *v/v*% methanol or acetonitrile in sodium phosphate (50 mM, pH 3.0), sodium acetate (50 mM, pH 5.0), and Tris-HCl (50 mM, pH 7.0) buffers (λ_ex_ = 345 nm).

The effects of detergents on the emission signal of AOH were also tested employing SDS and T-X100. Samples contained AOH (5.0 μM) and increasing concentrations of SDS (0.0, 0.5, 1.0, 2.0, 3.0, 5.0, 10.0, and 20.0 mM) or T-X100 (0.0, 0.2, 0.3, 0.4, 0.5, 1.0, 2.5, and 5.0 mM). Emission spectra were recorded using 345 nm excitation wavelength in sodium phosphate (50 mM, pH 3.0), sodium acetate (50 mM, pH 5.0), and Tris-HCl (50 mM, pH 7.0) buffers.

We tested the effects of different alkali and alkaline metal ions as well as transitional metal ions on the fluorescence signal of AOH: Increasing concentrations of Na^+^, K^+^, Ca^2+^, Mg^2+^, Ba^2+^, Zn^2+^, Fe^2+^, and Cu^2+^ (0, 50, 100, 200, 300, 500, and 1000 mM) were added to AOH (5.0 μM) in Tris-HCl buffer (50 mM, pH 7.0). Since Mg^2+^ proved to be a potent fluorescence enhancer of the mycotoxin, AOH-Mg^2+^ interaction was also examined at pH 3.0 (50 mM sodium phosphate buffer) and pH 5.0 (50 mM sodium acetate buffer). Emission spectra were recorded using 345 nm excitation wavelength. Binding constants of AOH-cation complexes were calculated employing non-linear fitting by the Hyperquad2006 software [27,28] as well as using the graphical application of the Benesi-Hildebrand equation [29,30].

Data presented are derived at least from three parallel, independent experiments. SEM (standard error of the mean) values did not exceed 5.1%.

### 3.3. Modeling Studies

According to our previous work [10], the molecular modelling studies have been performed at semi-empirical level using HyperChem 8 code. However, considering the presence of Ca^2+^ and Mg^2+^ ions, PM3 method is applied for both the geometry optimization and the vibrational-rotational analysis to calculate the thermodynamic parameters of the molecular interactions. The aqueous environment was considered by the TIP3P method. Both the enthalpy and entropy terms were calculated on the common way: the enthalpy change of the complex formation considered as the energy change calculated by subtracting the total energies of the reactants from the total energies of the products, while the entropy changes were calculated by subtracting the entropy terms of the reactants from the entropy terms of the products. Vibrational frequencies to calculate the vibrational entropy term are considered in harmonic approximation.

### 3.4. HPLC Analyses

The integrated HPLC system (Jasco, Tokyo, Japan) used contained an autosampler (AS-4050), a binary pump (PU-4180), and a fluorescence detector (FP-920). Chromatographic data were evaluated employing ChromNAV2 software (Jasco, Tokyo, Japan). The effects of Mg^2+^ on the sensitivity regarding the HPLC-FLD analyses of AOH were tested employing the following two methods. In the first HPLC method, a SecurityGuard guard column (C8, 4.0 × 3.0 mm; Phenomenex, Torrance, CA, USA) was linked to a Kinetex C8 (100 × 4.6 mm, 5 μm; Phenomenex, Torrance, CA, USA) analytical column; while in the second method, a SecurityGuard guard column (C18, 4.0 × 3.0 mm; Phenomenex, Torrance, CA, US) was linked to a Gemini C18 (150 × 4.6 mm, 5 μm; Phenomenex, Torrance, CA, US) chromatographic column. In both assays, the mobile phase consisted of HEPES buffer (10 mM, pH 7.0) and acetonitrile (70:30 *v*/*v*%), without or with MgCl_2_ (50 mM). The injected sample volumes were 20 μL, the analyses were carried out at 1.0 mL/min flow rate and room temperature. AOH was detected at 455 nm emission wavelength (λ_ex_ = 345 nm). The linearity was tested between 0 and 1000 nM mycotoxin concentrations. LOD and LOQ values were determined as the lowest concentrations where signal-to-noise ratios were 3 and 10, respectively. Intra-day repeatability was tested only for the Mg^2+^-sensitized methods (n = 7).

## 4. Conclusions

In summary, the effects of the microenvironment on the fluorescence properties of AOH have been examined. The environmental pH can strongly affect the excitation and emission spectra of the mycotoxin (Figure 2). In acetonitrile and methanol, the emission signal of AOH is much higher (approximately ten- and fivefold, respectively) than in water; however, in aqueous buffers, 20 and 50 *v*/*v*% concentrations of these organic solvents caused only minor increases (Figure 3). Detergents (20 mM of SDS and 5 mM of T-X100) caused approximately eight- to ten-fold increase in the fluorescence of AOH at pH 7.0 (Figure 4). Similarly, Ca^2+^ (1000 mM) induced a 12-fold elevation in the emission signal of the mycotoxin (Figure 5). However, Mg^2+^ forms more stable complexes with AOH than Ca^2+^ (Table 1 and Table 2), and causes a major (80-fold) increase in the emission intensity of the mycotoxin at pH 7.0 (Figure 6). Finally, we demonstrated that Mg^2+^ is suitable for the sensitization of the analytical detection of AOH by HPLC-FLD methods (Figure 8).

## Figures and Tables

**Table 1:** Binding constants (*K*) of AOH-cation complexes in Tris-HCl buffer (50 mM, pH 7.0).

Complex	*K* (L/mol) ± SEM Hyperquad	*K* (L/mol) ± SEM Benesi-Hildebrand
AOH-Mg^2+^	5.86 ± 0.62	4.11 ± 0.61
AOH-Ca^2+^	1.49 ± 0.04	1.75 ± 0.03

**Table 2:** Binding constants (*K*) of AOH-cation complexes based on modeling studies.

AOH	Interacting Ion	*K* (L/mol)
nonionic	Mg^2+^	5.54
monoanionic	Mg^2+^	5.91
nonionic	Ca^2+^	1.80
monoanionic	Ca^2+^	4.80

**Table 3:** LOD and LOQ values of the HPLC-FLD methods applied, without and with 50 mM MgCl_2_ in the eluent.

	LOD		LOQ	
Method	nM	μg/L	nM	μg/L
C8 without Mg^2+^	50	12.9	100	25.8
C8 with Mg^2+^	10	2.6	20	5.2
C18 without Mg^2+^	100	25.8	200	51.6
C18 with Mg^2+^	20	5.2	50	12.9

## Data Availability

Not applicable.
